# The rhizosphere of a drought‐tolerant plant species in Morocco: A refuge of high microbial diversity with no taxon preference

**DOI:** 10.1111/1758-2229.13254

**Published:** 2024-05-09

**Authors:** Jean Legeay, Khaoula Errafii, Abdelhadi Ziami, Mohamed Hijri

**Affiliations:** ^1^ African Genome Center University Mohammed VI Polytechnic (UM6P) Ben Guerir Morocco; ^2^ Institut de Recherche en Biologie Végétale Département de Sciences Biologiques, Université de Montréal Montreal Quebec Canada

## Abstract

Arid and semi‐arid areas are facing increasingly severe water deficits that are being intensified by global climate changes. Microbes associated with plants native to arid regions provide valuable benefits to plants, especially in water‐stressed environments. In this study, we used 16S rDNA metabarcoding analysis to examine the bacterial communities in the bulk soil, rhizosphere and root endosphere of the plant *Malva sylvestris* L. in Morocco, along a gradient of precipitation. We found that the rhizosphere of *M. sylvestris* did not show significant differences in beta‐diversity compared to bulk soil, although, it did display an increased degree of alpha‐diversity. The endosphere was largely dominated by the genus *Rhizobium* and displayed remarkable variation between plants, which could not be attributed to any of the variables observed in this study. Overall, the effects of precipitation level were relatively weak, which may be related to the intense drought in Morocco at the time of sampling. The dominance of *Rhizobium* in a non‐leguminous plant is particularly noteworthy and may permit the utilization of this bacterial taxon to augment drought tolerance; additionally, the absence of any notable selection of the rhizosphere of *M. sylvestris* suggests that it is not significatively affecting its soil environment.

## Graphical abstract

*Malva sylvestris*, a widely distributed plant thriving in arid and semi‐arid environments, harbours a substantial presence of nitrogen‐fixing *Rhizobium* bacteria within its roots. The bacterial community in its rhizosphere showed no significant variance compared to the adjacent bulk soil, indicating minimal impact of *M. sylvestris* on its soil environment.

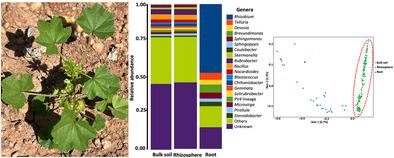

## INTRODUCTION

Morocco is characterized by a diverse range of topographies, including mountains, plateaux, plains, oases and Saharan dunes. Due to this variety, the country experiences diverse climatic conditions, featuring large spatial and intra‐ and inter‐annual variability in precipitation. As a result, Morocco has been facing irregular rain patterns, heat waves and period of cold weather, resulting in increasingly frequent droughts and damaging impacts on vegetation (Giustarini et al., 2023).

The year 2022 has demonstrated a record number of drought episodes around the world. It is becoming apparent that droughts will be a growing challenge in the coming years. To mitigate the effects of increasing droughts, studying plants and their associated microbiomes which can grow in dry environments is essential for the improved tolerance and resilience. A study conducted by Rolli et al. demonstrated that bacteria colonizing the roots of grapevines can offer protection against drought stress (Rolli et al., 2015). Bacteria can protect plants from drought in various ways (Zhang et al., 2022). A study on maize reported by Moore et al. (2023) showed that the beneficial microbial assemblages differed significantly among different water‐limited regimes (Moore et al., 2023). The microbiome of drought‐resistant plants also holds potential for helping to alleviate the effects of increasing aridity on other plants. For example, Schmitz et al. demonstrated that tomato grown in a soil microbiome containing a synthetic bacterial community from a desert rhizosphere showed increased tolerance to salt and drought stress (Schmitz et al., 2022). Unfortunately, Africa and the Middle East—the two inhabited continents with the highest percentage of arid areas (33% and 80%, respectively)—have not been sufficiently studied at the microbiome level and may well reveal many interesting discoveries about arid microbiomes and potentially relevant microbial species.

Some studies have investigated the core microbiome of plants growing in arid conditions capable of withstanding extreme drought. Khan et al. (2022) examined the halophyte shrub *Zygophyllum qatarensis* and found no core bacterial or fungal microbiome associated to the plant. In a study of two Mexican *Cactaceae* species, Fonseca‐García et al. (2016) did not find any core microbiome in either species and microbial communities did not show host specificity. Interestingly, Araya et al. (2020) determined a small core microbiome consisting of six operational taxonomic units in a desert annual plant. Additionally, Coleman‐Derr et al. (2016) identified a core microbiome in the endosphere of three agave species in Mexico and California, which was more expanded during the dry season of the year. However, these studies only focused on plants found naturally in arid environments. Given the expected increase in the number of extreme weather events, it is important to study the microbiome of a wild plant that is capable of withstanding long periods of drought but which also occurs in more temperate climates.

*Malva sylvestris* L. is a plant in the Malvaceae family, widely distributed across three continents from China to Morocco and along an important gradient of aridity, demonstrating resistance to both drought and heavy rain (Cossel, 2020). Growing virtually in any kind of soil, its nutritional value has been described as similar or superior to that of spinach (Basheer et al., 2021). Historically, many cultures have eaten this plant, while their medicinal properties have been used since antiquity (Bouayyadi et al., 2015; Gasparetto et al., 2012). Additionally, *M. sylvestris* extracts have been shown to possess antibacterial activities (Razavi et al., 2011).

In this study, we investigated the bacterial community of the plant *M. sylvestris* in both agricultural and natural environments to determine the structure of microbiome of this plant, particularly in arid regions and evaluate which bacteria are associated with this plant's drought tolerance. We hypothesize that *M. sylvestris* plants from arid environments select a different microbiome than those from high precipitation levels. To test this hypothesis, we sampled 101 individuals from 13 sites across Morocco, which included cultivated and non‐cultivated areas along a gradient of precipitation. 16S rDNA metabarcoding was used to investigate bacterial communities in the bulk soil, rhizosphere and root endosphere of *M. sylvestris*.

## EXPERIMENTAL PROCEDURES

### Sampling

**FIGURE 1:**
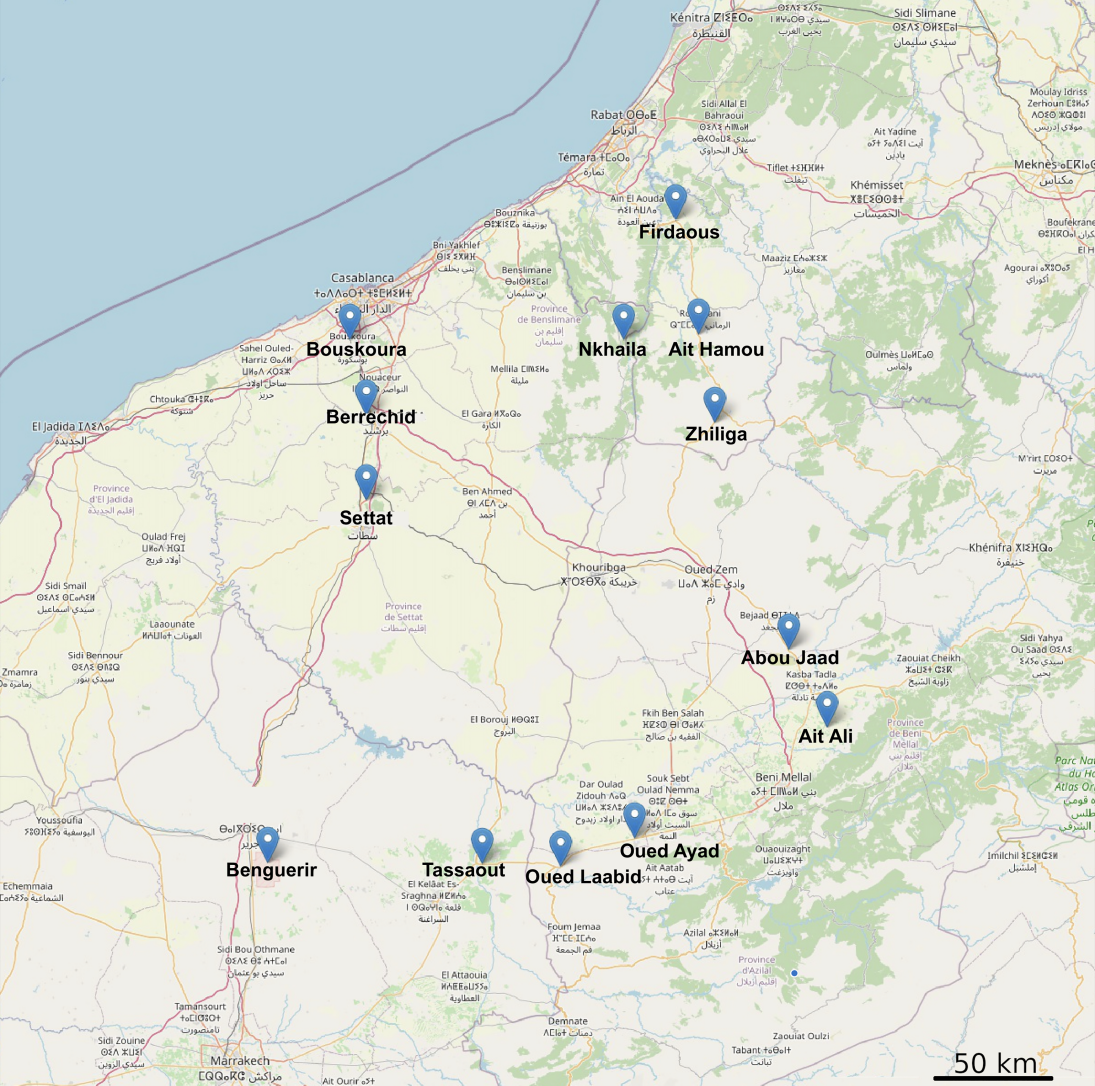
A map showing the 13 sites that have been sampled in Morocco. The scale bar indicates a distance of 50 km (created using the R *leaflet* package).

Samples of *M. sylvestris* were taken in 13 sites across Morocco, including both cultivated and non‐cultivated areas (Figure 1). The sampling campaign was on 21 February 2022 and 22 February 2022, which correspond to the period of vegetative growth before flowering. These sites were selected to represent a latitudinal aridity gradient from 290 to 530 mm/year, as well as to reflect various longitudes to distinguish aridity from geographic distance. At each site, between five and six individuals of *M. sylvestris* plants were collected by taking the whole plant with its associated soil. In some sites where different plants were cultivated in more than one culture, the sampling has been repeated in the different crops (Table S1). Since two distinct phenotypes, erected and creeping/spread, were observed in the field, the samples were classified accordingly. Bulk soil samples were also gathered from five points in each location. The samples were placed inside a plastic bag then stored in an icepack‐cooled container during the sampling period.

At the laboratory, the shoots were cut away from the roots. Each individual's shoots were put into paper bags and dried for 24 h in an oven at 70°C and then weighted. The roots were shaken to acquire and collect the rhizosphere particles which were stored in plastic bags. The roots were washed with tap water to eliminate soil particles, then rinsed with sterile demineralized water and dried on paper towels at ambient temperature and then lyophilized. All samples were stored at −20°C for 3 months until use.

### Soil physicochemical analyses

An equal amount of bulk soil and rhizosphere soil was blended at each site to create composite samples. These composite samples were sent to the AITTC of the University Mohammed VI Polytechnic (Benguerir, Morocco) for physicochemical analysis (Table S1).

### DNA extraction, amplification and sequencing

DNA from soils was extracted using Qiagen PowerSoil Pro and DNA from roots was extracted with Qiagen DNeasy Plant Pro, according to the manufacturer's instructions (Qiagen, Global Diagnostic Distribution, Temara, Morocco). Fifty micrograms of lyophilized root material were ground using a Qiagen TissueLyzer, then used for DNA extraction. For soil samples, 250 mg of soil were used for extraction. The final elution volume for both DNA extractions was 50 μL. The bacterial V3 and V4 regions of the 16S was then amplified using the high‐performance liquid chromatography‐purified primers CS1/341F (5′‐ACACTGACGACCATGGTTCTACACCTACGGGN‐3′) and CS2/806R (5′‐ TACGGTAGCAGAGACTTGGTCTGACTACHVG‐3′) (Alpha DNA, Montreal, Canada), using a thermocycler with an initial denaturation of 94°C for 3 min, followed by 30 cycles of 94°C for 30 s, 55°C for 30 s and 72°C for 1 min, with a final elongation step at 72°C for 7 min. Libraries were prepared as follow: AMPure XP beads (Beckman Coulter, Rabat, Morocco) were added, mixed and separated magnetically. Two ethanol washes were performed, followed by air‐drying. DNA was then resuspended in 10 mM Tris (pH 8.5). Following the initial purified PCR amplification of target regions of 16S bacterial rDNA, a secondary PCR reaction was carried out using unique indexing primers (Fluidigm, Markham, ON, Canada) for each sample. This step allowed for the attachment of sample‐specific indexes and adapters to the amplicons, enabling multiplexing of samples for sequencing. The indexed amplicons were subsequently purified using ampure XP beads, quantified using Qubit assay using DNA HS kit (Thermo‐Fisher Scientific, Rabat, Morocco) and pooled prior to sequencing, thus enabling the parallel analysis of diverse bacterial communities in the samples. Sequencing was then performed on a MiSeq instrument utilizing MiSeq Reagent Kit V3 (600‐cycles) (Illumina, MegaFlex, Casablanca, Morocco).

### Bioinformatics data processing and statistical analysis

BCL to fastq conversion was performed in MiSeq Reporter using bcl2fastq pipeline.

These raw reads were analysed through the DADA2 pipeline implemented in R version 4.3.1 (Callahan et al., 2016). Reads with an expected error rate of more than two were discarded, and forward and reverse reads were truncated at 50 and 100 bp, respectively, due to low quality at those ends. Using the consensus method, Chimera sequences were discarded. The resulting amplicon sequence variants (ASVs) were taxonomically assigned using SILVA database (Quast et al., 2012) and ‘assignTaxonomy’ command's default parameters.

The reads corresponding to chloroplastic DNA, amounting to 85% of reads in the root samples, were discarded. The raw data were transformed into compositional data using the *microbiome* package in R (Lahti & Shetty, 2017). Then, the alpha diversity was calculated by the Shannon and Simpson indices, using the estimate_richness command from the *phyloseq* package (Paul et al., 2017). Statistical differences were then determined through analysis of variance (ANOVA).

Beta‐diversity was calculated using the Bray–Curtis distance between samples and then plotted via principal coordinate analysis (PCoA) using the *vegan* package (Oksanen et al., 2022). Statistical tests were then conducted using the ADONIS PERMANOVA. ANOVA was performed for canonical‐correlation analysis to investigate the correlation between quantitative factors and communities. An analysis of the best ASVs for predicting precipitation levels was conducted utilizing the randomForest package (Breiman et al., 2022), with the parameter ntree = 100, after discarding all ASVs with less than 5% prevalence to reduce the dataset size for computing reasons. Additionally, a differential abundance analysis was carried out using the ANCOMBC package (Lin & Peddada, 2020), also on ASVs with more than 5% prevalence.

## RESULTS

### Soil and plant characteristics

The measurements of total amounts of nitrogen (N), carbon (C), phosphorus (P) and potassium (K) are provided in Table S1. A strong correlation (*p* = 1.49e‐06 and *p* = 2.54e‐06, respectively) was found between K and P concentrations in agricultural soils, with a higher amount of K and P in agriculturally utilized soils. However, no significant correlation was found between total C and N concentrations and agricultural use of the field (*p* = 0.84 and *p* = 0.60, respectively). There were no statistical differences between rhizosphere and bulk soil chemical characteristics. Furthermore, a higher likelihood of the presence of an erected phenotype was seen in regions with higher precipitation levels (*p* < 0.001) and higher amounts of total C (*p* = 0.02).

### Taxonomic composition

**FIGURE 2:**
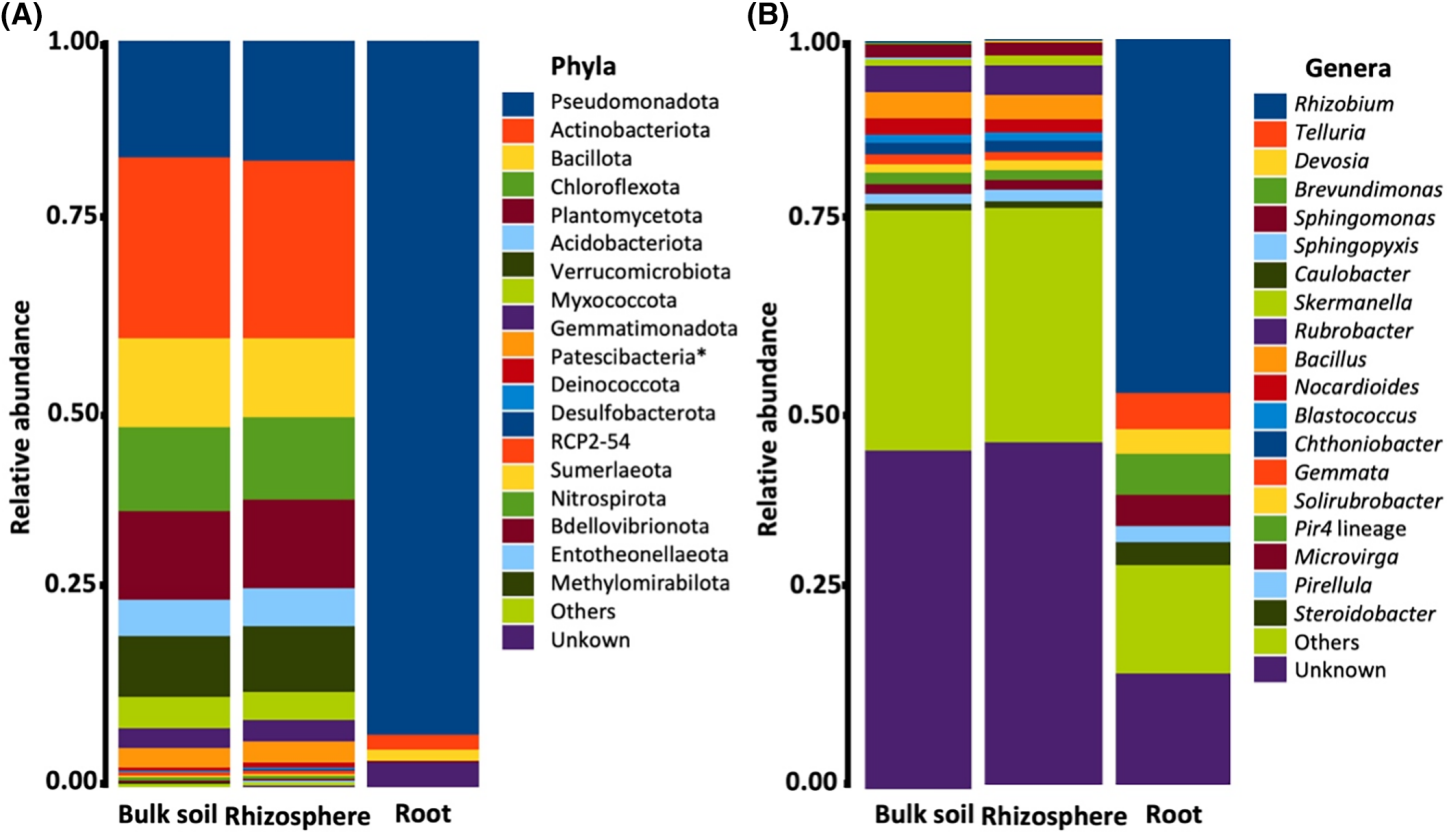
The relative abundances of the 20 most common taxa (A, Phyla; B, Genera) are shown with all other taxa grouped as ‘Others’. The asterisk (*) denotes that Patescibacteria is categorized not as a phylum but as a group known as candidate phyla radiation (CPR).

**FIGURE 3:**
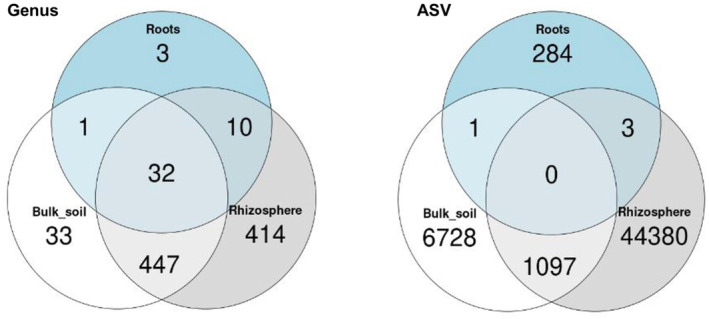
Venn diagrams illustrating the taxa of both genus (left panel) and amplicon sequence variants (ASV) (right panel) found in the different biotopes.

In total, 52,380 bacterial ASVs were obtained, classified into 937 genera, 397 families, 281 orders, 124 classes and 47 phyla. Rarefaction curves showed that most samples have attained saturation, except for two samples in the roots (Figure S1). The most abundant phyla in the soil samples were *Actinomycetota* (24%), *Pseudomonadota* (16%), *Planctomycetota* (12%), *Chloroflexota* (11%) and *Bacillota* (11%). The dominant phyla in the root endosphere were *Pseudomonadota* (91%), with bacteria of unknown phyla representing 4% of reads (Figure 2). The most abundant genus in the roots was *Rhizobium*, representing 48% of reads assigned to ASVs. The most abundant genera in the soils in agricultural fields were *Rubrobacter* (5%), *Bacillus* (4%), *Microvirga* (2%), *Sphingomonas* (2%), *Solirubrobacter* (1%) (Figure 2). The most abundant genera in the soils of uncultured fields were *Nocardioides* (3%), *Bacillus* (3%), *Rubrobacter* (2%), *Sphingomonas* (2%), and *Pir4* lineage (2%) (Figure 2). There were substantially more reads of an unknown genus in the soil samples (46% in the rhizosphere and 45% in the bulk) than in the endosphere (10%). Moreover, most ASVs were specific to a single biotope, with no ASV observed in all three biotopes (Figure 3). At the genus level, however, there were more common taxa; 31 genera were shared between all biotopes, while 447 were shared between rhizosphere and bulk soil (Figure 3).

### Alpha‐ and beta‐diversities

Alpha‐diversity indexes in soil samples were much higher than those in the endosphere (Table 1). The bulk and rhizosphere soil samples also displayed a difference in alpha‐diversity, with the rhizosphere samples being significantly more diverse (*p* = 8.98e‐06 and *p* = 5.21e‐06). The standard deviation for endosphere samples was much greater than for soil samples.

**TABLE 1:** Shannon and Simpson indices in the different compartments.

Compartment	Shannon	Simpson
Bulk soil	5.6 ± 0.2	1 ± 0.00
Rhizosphere	5.8 ± 0.1	1 ± 0.00
Roots	1.1 ± 0.7	0.51 ± 0.29

In both the roots endosphere and the rhizosphere, a Kruskal–Wallis test of Shannon and Simpson diversity indexes showed no differences between agricultural and uncultivated sites, plant phenotypes, precipitation levels or total C, N, K, or P (Table 2). Overall, both in the roots endosphere, no factor was found to significantly influence alpha‐diversity.

**TABLE 2:** Statistical significance (*p*‐values) of the different factors on the Shannon and Simpson indices, in the rhizosphere and roots compartment, according to analysis of variance (ANOVA) for qualitative factors or Pearson correlation test for quantitative factors.

Factor	Compartment	Shannon	Simpson
Total P	Rhizosphere	0.16	0.09
	Roots	0.212	0.205
Total C	Rhizosphere	0.95	0.88
	Roots	0.792	0.761
Total K	Rhizosphere	0.55	0.62
	Roots	0.07	0.06
Total N	Rhizosphere	0.4	0.25
	Roots	0.37	0.28
Precipitation	Rhizosphere	0.1	0.05
	Roots	0.21	0.23
Agricultural use	Rhizosphere	0.74	0.5
	Roots	0.495	0.498
Crop	Rhizosphere	0.18	0.12
	Roots	0.04	0.048

**FIGURE 4:**
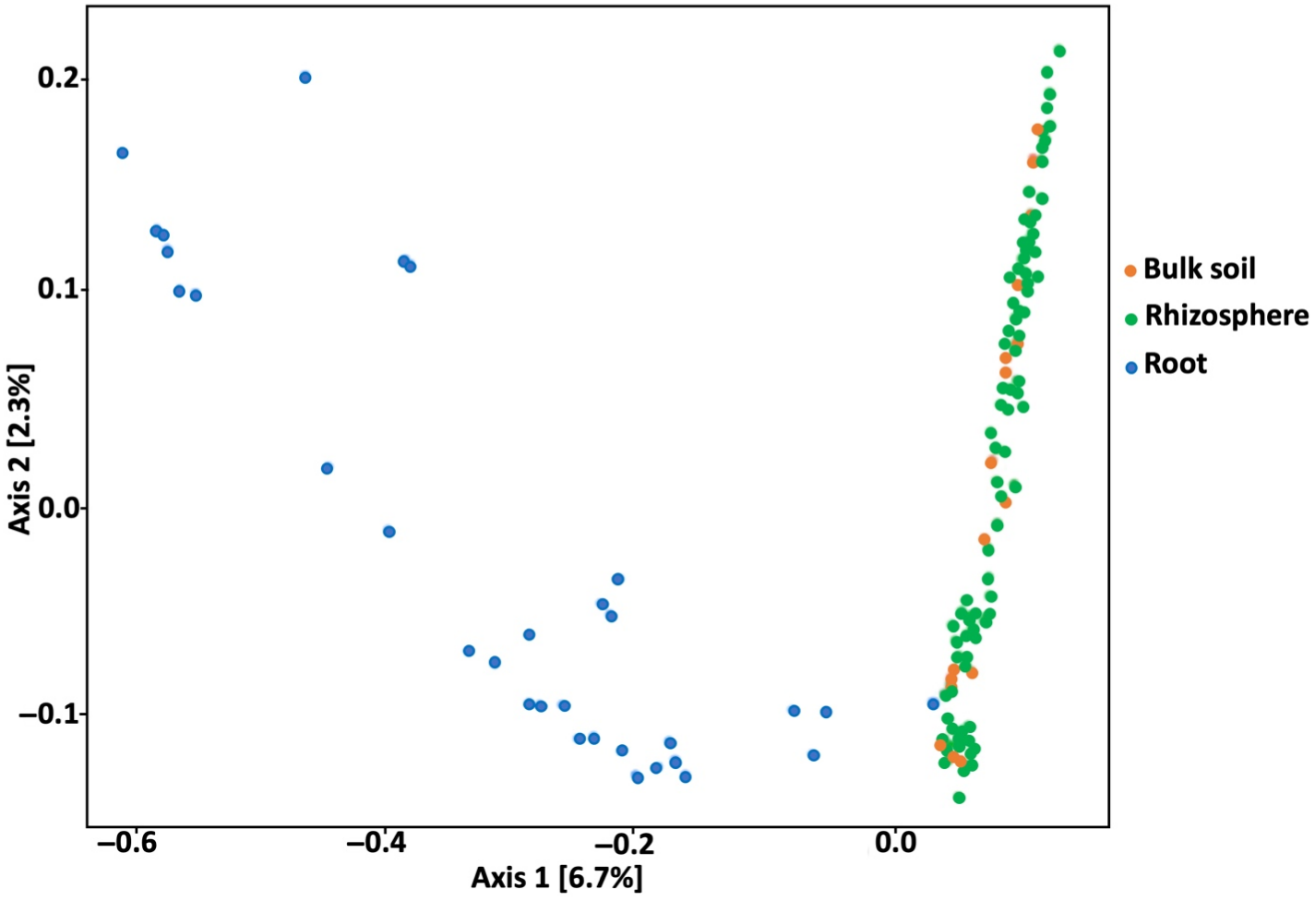
Principal coordinate analysis (PCoA) plot of the samples, grouped by their respective biotope.

The results for the beta‐diversity analysis did not mirror the alpha‐diversity results, as no significant differences were detected between the bulk and rhizosphere bacterial profiles by PERMANOVA. However, the endosphere differed significantly from the soil samples (*p* < 0.001) (Figure 4). Factors such as agricultural practice of the site (*p* < 0.001) and crop cultivated on the site (*p* < 0.001) explained the beta‐diversity between the soil communities (Table 3). ‘*betadispers* function’ showed a higher level of dispersion in the endosphere samples (0.654) than bulk and rhizosphere samples (0.677 and 0.687, respectively). Soil parameters (total P, total N, total C and total K) and precipitation volume were significant according to the ANOVA practiced on CCA results (Figure S2). The phenotype and dry weight of the plants did not affect rhizosphere and endosphere bacterial communities.

**TABLE 3:** *p*‐values and chi‐square value (if the *p*‐value is significative) of each factor in the beta‐diversity of the community. As the beta‐diversity between bulk soil and rhizosphere was not significant, they were regrouped under soil samples.

Factor	Compartment	*p*‐value	Chi‐square (%)
Total P	Soil	0.001***	1.00
	Roots	0.318	
Total C	Soil	0.028**	1.00
	Roots	0.412	
Total K	Soil	0.001***	1.40
	Roots	0.546	
Total N	Soil	0.001***	1.50
	Roots	0.569	
Precipitation	Soil	0.001***	1.20
	Roots	0.568	
Agricultural use	Soil	0.001***	2
	Roots	0.885	
Crop	Soil	0.001***	2
	Roots	0.905	
Plant phenotype	Soil	0.802	
	Roots	0.677	
Plant dry weight	Soil	0.991	
	Roots	0.557	

The Mantel test failed to find any influence of geographic distance between sites on either the soil (*p* = 0.198) or root (*p* = 0.774) bacterial communities.

### Response of taxa to environmental factors

The ASV52429, associated with the *Rhizobium* genus and located in the roots, demonstrated a statistically significant positive correlation with the plants' weight (*p* < 0.001). Additionally, three ASVs from the roots exhibited positive correlations with precipitation levels, comprising two from the *Rhizobium* genus and one from the *Devosia* genus (*p* = 0.006 for each ASV). Lastly, ASV1 from the soil exhibited a negative correlation with the total phosphorus level (*p* = 0.008). The Rhizobiales order itself did not show a correlation with precipitation levels (*p* = 0.25).

Among the genera that experienced a relative abundance increase due to agricultural practice were *Nitrospira*, *Rubrobacter*, *Microvirga*, *Solibacillus* and *Bacillus*; these genera were among the most abundant in the community and were most closely associated with agricultural fields in arid environments. Those that experienced a decrease in abundance included *Marmoricola*, *Nocardioides*, *Luteitalea*, *Kocuria* and *Paracoccus*. Precipitation level was less significant on the genus composition (*p* = 0.02) but affected the genera most associated with high precipitation, such as *Luedemannella*, *Lysobacter*, *Actinomadura*, *Actinoallomurus* and *Terrimonas*.

At the phylum level, precipitation volumes, total P, total K and total N were insignificant (*p* > 0.1 in every case). Only agricultural practice of the site (*p* = 0.005) and the total C (*p* = 0.01) remained significant. *Bacillota* and *Acidobacteriota* were positively correlated to agricultural practice and *Pseudomonadota* and *Chloroflexota* were negatively correlated to it.

Following a trimming process to retain only ASVs representing more than 0.1% of the total sum of abundance, resulting in 923 remaining ASVs, a random forest model was employed to identify optimal predictors for both agricultural practice and precipitation levels. When constructing a model to distinguish samples from semi‐arid (annual precipitations <300 mm) and sub‐humid (>300 mm) regions, 29 out of 160 samples were misclassified. Notably, 6 of the top 20 predictor ASVs belonged to the Rhizobiales order (Table 4). In differentiating between agricultural and uncultivated areas, 53 out of 160 samples were misclassified. The list of the 20 most important taxa for predictions can be found in Table S2.

**TABLE 4:** Twenty amplicon sequence variants (ASVs) with the greatest importance for predicting the precipitation level of the sampled field according to a random forest model.

ASV	Order	Family	Genus	Relative abundances		Correlation to precipitation	
				Dry	Humid	*r*	*p*
52411	Rhizobiales	Rhizobiaceae	*Rhizobium*	2.59E‐02	2.02E‐02	−0.205806	0.83
52642	Burkholderiales	Oxalobacteraceae	*Telluria*	0.00E+00	1.67E‐03	0.7177459	0.47
52428	Caulobacterales	Caulobacteraceae	*Brevundimonas*	0.00E+00	3.03E‐02	0.7177459	0.47
52447	Rhizobiales	Rhizobiaceae	*Rhizobium*	0.00E+00	3.03E‐02	0.7177459	0.47
52710	Burkholderiales	Comamonadaceae	*Aquincola*	0.00E+00	3.03E‐02	2.4410141	0.016
53	Bacillales	Bacillaceae	Unknown	2.49E‐03	1.17E‐02	3.3827864	9.06E‐04
477	Bacillales	Bacillaceae	*Bacillus*	8.55E‐05	8.55E‐05	4.5253065	1.18E‐05
52570	Enterobacterales	Yersiniaceae	Unknown	0.00E+00	3.30E‐03	2.4410141	0.016
252	Bacillales	Unknown	Unknown	0.00E+00	8.09E‐03	2.9814974	3.33E‐03
52711	Burkholderiales	Oxalobacteraceae	*Telluria*	0.00E+00	3.03E‐02	2.4410141	0.016
52531	Pseudomonadales	Pseudomonadaceae	Unknown	0	1.75E‐02	0.7177459	0.47
209	Micromonosporales	Micromonosporaceae	*Luedemannella*	0	5.78E‐03	5.832338	3.02E‐08
52596	Rhizobiales	Rhizobiaceae	Unknown	4.64E‐04	2.37E‐03	0.4015999	0.69
52490	Rhizobiales	Rhizobiaceae	*Rhizobium*	0.00E+00	1.62E‐02	2.4410141	0.016
52601	Enterobacterales	Unknown	Unknown	0.00E+00	7.58E‐03	0.7177459	0.47
52408	Rhizobiales	Rhizobiaceae	*Rhizobium*	5.27E‐02	4.79E‐02	0.0478632	0.96
52551	Rhizobiales	Rhizobiaceae	Unknown	0.00E+00	1.41E‐02	0.7177459	0.47
163	Rhizobiales	Xanthobacteraceae	Unknown	2.94E‐04	5.18E‐03	3.3551422	9.95E‐04
52452	Sphingomonadales	Sphingomonadaceae	*Sphingomonas*	0	2.43E‐02	0.7177459	3.36
52575	Caulobacterales	Caulobacteraceae	*Asticcacaulis*	2.85E‐04	0.00E+00	−0.481133	0.63

The ANCOMBC analysis identified eight ASVs with a positive log fold change for each incremental rise in precipitation levels, along with seven ASVs displaying a negative log fold change. Nevertheless, none of these ASVs successfully passed the sensitivity analysis test, indicating a potential bias in the results due to the introduction of pseudo‐counts during the application of the ANCOM method (Figure S3).

### Core taxa

No core ASV was identified in more than 80% of the rhizosphere samples. However, at the genus level, 32 genera were identified in more than 80% of the rhizosphere samples. These 32 ‘core genera’ accounted for 75% of the reads in the rhizosphere. However, none of these taxa was significantly correlated to the rhizosphere. No taxon was significantly associated with the rhizosphere at all, suggesting that all taxa present in the rhizosphere were also present in the bulk soil. In the endosphere samples, ASVs related to the genus *Rhizobium* were present in 80% of the samples and the presence of *Rhizobium* was significantly correlated to the root endosphere. None of the *Rhizobium* ASVs could be confidently linked to known species within the *Rhizobium* genus.

## DISCUSSION

Regarding the impact of aridity levels and agricultural practice on the bacterial community, both the PERMANOVA analysis and the random forest model analysis identified taxa associated with specific conditions. However, the random forest model exhibited weaker predictive power in discriminating between agricultural practice. Interestingly, certain ASVs appeared important for predicting aridity, irrigation and showing significant correlations with precipitation belong to the Rhizobiales order, a topic that will be discussed further.

Additionally, ASVs involved in predicting precipitation levels include those associated with the genera *Telluria*, *Brevundimonas* and *Aquincola* in the roots, as well as *Bacillus* in the soil. Among the 20 most predictive ASVs for aridity, only 5 are predominantly found in the soil, while the majority are concentrated in the roots. Similarly, ASVs most correlated with precipitation all originate from the roots. This observation could potentially be attributed to the fact that during the sampling period, Morocco experienced an unusually severe drought episode, which might have homogenized drought conditions in the soil. In contrast, the root community may have retained its adaptation for a longer duration.

Surprisingly, *M. sylvestris* did not exhibit a core microbiome significantly associated with its rhizosphere. As mentioned in the introduction, the core microbiomes of plants thriving in arid conditions often tend to be very small or non‐existent. The absence of differences in beta‐diversity between the bulk soil and rhizosphere suggests that the rhizosphere bacterial community is not influenced or selected by *M. sylvestris*. This finding aligns with Khan et al. (2020), who observed a significant difference in beta‐diversity among rhizospheres of medicinal plants in Omani soils.

Therefore, it appears that *M. sylvestris* does not significantly shape its rhizosphere through the release of exudates or other mechanisms. These results contradict our hypothesis, which was subsequently rejected. Similar findings were reported by Naylor et al. (2017) in Poaceae species in California, where drought was found to decrease the host specificity of plant microbiomes. The lack of selection observed in our study may be attributed to the fact that plants experiencing intense drought stress may not have the resources to allocate for the production of exudates to shape the microbiome in their rhizosphere, as hypothesized by Khan et al. (2022).

*M. sylvestris* has been described in Brown et al. (2017) as having a strong rhizosheath: an agglomeration of soil particles surrounding the roots that is highly resistant to drought. Additionally, the rhizosheath has been found to harbour bacteria that can aid the plant in overcoming the various problems associated with drought, such as the reduced availability of nutrients from the soil (Etesami, 2021). Marasco et al. (2018) showed that the bacterial assemblages in the rhizosheaths of desert speargrass were recruited from the bulk soil and were enriched when compared to the surrounding bulk soil, creating an ‘edaphic mini‐oasis’, rich in plant growth‐promoting bacteria (Marasco et al., 2022). Liu et al. (2021) contradict this result in their study on switchgrass, instead showing that rhizosheath bacterial community was selected by the host plant. Contrary to what is usually observed in temperate or boreal forest areas, or other desert areas (Araya et al., 2020; Fonseca‐García et al., 2016), the alpha‐diversity of the rhizosphere of *M. sylvestris* was slightly higher than the bulk soil. This suggests that the rhizosphere of *M. sylvestris* acts more as a shelter from the arid and hot conditions of the bulk soil, a hypothesis that may be even stronger at the rhizosheath level. Consequently, a more specific study of the microbiome of the *M. sylvestris* rhizosheath biotope is needed.

*M. sylvestris* exhibits a difference in phenotypic adaptation between two aridity regions, with an erect phenotype being more common in high‐precipitation areas and a spread phenotype dominating in low‐precipitation regions. However, there is no clear indication of selection of bacteria in the rhizosphere, nor is there a correlation between the plant microbiome and phenotype or dry weight, indicating that the microbiome of *M. sylvestris* does not actively contribute to its adaptation to the environment, at least not on the morphological level.

The *M. sylvestris* endosphere microbiome showed high dispersal and variability in alpha‐diversity among samples, without any factor appearing to structure it. One hypothesis to explain this fact could be that a strategy of the plant to cope with drought stress is to increase its intake of bacteria in the roots. *Rhizobium* was the dominant genus in the endosphere and firmly associated with it, making is a core taxon to the *M. sylvestris* root endosphere microbiome. Although microbial genera found in endosphere could also be observed in the rhizosphere, there was little ASV‐level parallelism between the two biotopes. Consequently, the factors dictating the presence or absence of bacterial species in the *M. sylvestris* endosphere are still not well understood. The relative abundance of *Rhizobium* indicates that certain selective forces may be put in place, but it appears that these are mostly stochastic. Similar results were also reported in a study of desert agaves by Coleman‐Derr et al. (2016), who found that 40% of the bacterial community in the endosphere became a core microbiome. However, even that study could not find link with any biogeographic factor.

The ASVs that demonstrated significant predictive capability between aridity levels included seven Rhizobiales primarily located in the roots, predominantly associated with the *Rhizobium* and *Devosia* genera. However, only one of those predictive ASVs exhibited a significant correlation to precipitation. Thus, the predictive power may be attributed to localized effects rather than a simple association with precipitation. Although the overall relative abundance of Rhizobiales in the roots did not correlate with precipitation values, two specific *Rhizobium* ASVs showed strong positive correlations with precipitation.

Furthermore, another *Rhizobium* ASV displayed a positive correlation with the total dry weight of the plant, suggesting a potential modulation of Rhizobiales endophytes of *M. sylvestris* in response to environmental conditions, thereby influencing plant growth. Two *Rhizobium* ASVs also proved to be effective predictors of the agricultural practice in the sampling area, although with a somewhat less robust discrimination potential in the random forest model. This trend affirms the notable responsiveness of *Rhizobium* endophytes to environmental conditions.

The impact of *Rhizobium* symbiosis has predominantly been explored in leguminous plants capable of forming nodules. However, Barquero et al. (2022) and Mehboob et al. (2009) investigated its potential to enhance drought tolerance in non‐leguminous plants. In contrast to findings in other non‐legume plants inoculated with *Rhizobium* (Trinick & Hadobas, 1995), the presence of *Rhizobium* ASVs in the roots of *M. sylvestris* did not lead to the formation of nodules. As far as we know, the prevalence of *Rhizobium* hasn't been documented for a non‐legume plant, although agaves studied by Coleman‐Derr et al. (2016) and the root of desert plants investigated by Maurice et al. (2023) also showed a significant presence of *Rhizobiales*. As a result, the potential association of *M. sylvestris* with *Rhizobium* presents an intriguing avenue for exploring the nature of interaction between these *Rhizobium* taxa and both legume and non‐legume plants. This suggests the need for isolating and characterizing *Rhizobium* isolates from the roots of *M. sylvestris* and employing them as inoculants for both legume and non‐legume plants. Furthermore, other prevalent genera in the endosphere, such as *Sphingomonas* (Luo et al., 2019), *Devosia* (Moore et al., 2023) and *Phyllobacterium* (Bresson et al., 2013), have also demonstrated the ability to enhance drought tolerance.

*Actinomycetota* and *Pseudomonadota* were found to dominate the rhizosphere of *M. sylvestris* in this arid steppic environment of Morocco, consistent with findings from other arid environments. Despite the morphological adaptation of *M. sylvestris* to aridity, it did not appear to affect the bacterial community in its rhizosphere. This is an intriguing discovery since rather than selecting specific types of bacteria, it seems to promote diverse bacterial communities; this might be attributed to an evolutionary strategy of economizing resources by not exuding substances that can regulate the microbiome. The factors driving the bacterial communities of the root endosphere are still unknown; it is possible some of these, especially those of the dominant *Rhizobium* genus, might offer drought resistance.

## AUTHOR CONTRIBUTIONS

**Jean Legeay:** Data curation (equal); formal analysis (equal); investigation (equal); writing – original draft (equal). **Khaoula Errafii:** Data curation (equal); methodology (equal); writing – review and editing (equal). **Abdelhadi Ziami:** Methodology (equal). **Mohamed Hijri:** Conceptualization (equal); funding acquisition (equal); project administration (equal); supervision (equal); writing – review and editing (equal).

## CONFLICT OF INTEREST STATEMENT

The authors declare no conflicts of interest.

## Supporting information


**Data S1.** Supporting Information.

## Data Availability

Raw reads are available in the NCBI repository under the accessions SAMN31428469‐SAMN31428578 for the soil samples and SAMN37266206‐SAMN37266275 for the root samples.
