# Supplementary material for: The rhizosphere of a drought‐tolerant plant species in Morocco: A refuge of high microbial diversity with no taxon preference
Source: Environ Microbiol Rep. 2024 May 9;16(3):e13254. doi: 10.1111/1758-2229.13254 (PMC11082428; doi:10.1111/1758-2229.13254)
Supplement: Supplementary file 1 — Data S1. Supporting Information. [file EMI4-16-e13254-s001.docx]

Supplementary Information

Two tables: Tables S1 and Table S2.

Three figures: Figure Si, Figure S2 and Figure S3.

Table S1. Chemical and environmental features of the locations used for sampling.

| **Location** | **Crop** | **Latitude** | **Longitude** | **Precipitation** | **Total N** | **TotalP** | **Total K** | **Total C** |
| --- | --- | --- | --- | --- | --- | --- | --- | --- |
| Abou Jaad |  | 32.692 | -6.338 | 356 | 0.06 | 0.12 | 0.26 | 2.05 |
| Ait Ali | Pea | 32.496 | -6.2224 | 357 | 0.21 | 0.55 | 0.42 | 2.04 |
| Ait Ali | Beetroot | 32.496 | -6.2232 | 357 | 0.16 | 0.56 | 0.4 | 1.49 |
| Ait Ali | Faba Bean | 32.496 | -6.2227 | 357 | 0.14 | 0.48 | 0.37 | 1.79 |
| Ait Ali | Clover + Wheat | 32.496 | -6.2232 | 357 | 0.2 | 0.63 | 0.43 | 2.01 |
| Ait Hamou | Oignon | 33.488 | -6.611 | 528 | 0.17 | 0.07 | 0.57 | 1.66 |
| Ait Hamou | Oat | 33.488 | -6.611 | 528 | 0.18 | 0.06 | 0.64 | 2.08 |
| Benguerir |  | 32.15 | -7.907 | 291 | 0.18 | 0.09 | 0.48 | 1.7 |
| Berrechid |  | 33.285 | -7.612 | 343 | 0.16 | 0.31 | 0.4 | 2.14 |
| Bouskoura |  | 33.476 | -7.661 | 343 | 0.13 | 0.16 | 0.15 | 1.81 |
| Bouskoura | Barley | 33.476 | -6.836 | 343 | 0.18 | 0.09 | 0.16 | 1.77 |
| Firdaous |  | 33.476 | -6.836 | 528 | 0.06 | 0.05 | 0.05 | 0.69 |
| Nkhaila |  | 33.774 | -6.68 | 343 | 0.2 | 0.06 | 0.26 | 2.36 |
| Oued Laabid |  | 32.14 | -7.027 | 356 | 0.2 | 0.2 | 0.21 | 3.81 |
| Ouled Ayad |  | 32.212 | -6.8045 | 356 | 0.48 | 0.35 | 0.34 | 2.46 |
| Settat | Olive | 33.071 | -7.612 | 343 | 0.27 | 0.3 | 0.46 | 2.47 |
| Settat | Wheat | 33.071 | -7.612 | 343 | 0.31 | 0.57 | 0.92 | 2.96 |
| Tassaout |  | 32.148 | -7.262 | 356 | 0.23 | 0.07 | 0.26 | 2.2 |
| Zhiliga | Orange | 33.267 | -6.5621 | 420 | 0.25 | 0.1 | 0.16 | 2.08 |
| Zhiliga | Olive | 33.267 | -6.5621 | 420 | 0.25 | 0.1 | 0.16 | 2.08 |

Table S2. The list of the 20 most important taxa for predictions.

| **ASV** | **Order** | **Family** | **Genus** | **No** | **Yes** |
| --- | --- | --- | --- | --- | --- |
| **2** | Thermomicrobiales | JG30-KF-CM45 | Unknown | 0.0014 | 0.01023 |
| **5** | Rhizobiales | Beijerinckiaceae | Microvirga | 0.00246 | 0.00752 |
| **10** | Rubrobacterales | Rubrobacteriaceae | Rubrobacter | 0.00225 | 0.00478 |
| **12** | Thermomicrobiales | JG30-KF-CM45 | Unknown | 0.00503 | 0.00188 |
| **16** | Thermomicrobiales | JG30-KF-CM45 | Unknown | 0.00055 | 0.0045 |
| **18** | Azospirillales | Azospirillaceae | Skermanella | 0.0059 | 0.00109 |
| **30** | Rubrobacterales | Rubrobacteriaceae | Rubrobacter | 0.00103 | 0.00338 |
| **32** | Bacillales | Bacillaceae | Bacillus | 0.00147 | 0.00397 |
| **44** | Micrococcales | Micrococcaceae | Pseudarthrobacter | 0.00432 | 0.00095 |
| **102** | Rubrobacterales | Rubrobacteriaceae | Rubrobacter | 0.00057 | 0.00177 |
| **113** | Thermomicrobiales | JG30-KF-CM45 | Unknown | 0.00419 | 0 |
| **137** | Bacillales | Bacillaceae | Bacillus | 0.00037 | 0.00166 |
| **145** | Rubrobacterales | Rubrobacteriaceae | Rubrobacter | 0.00048 | 0.00153 |
| **151** | Bacillales | Bacillaceae | Bacillus | 0.00088 | 0.00143 |
| **156** | Thermomicrobiales | JG30-KF-CM45 | Unknown | 0.00242 | 0 |
| **203** | Micrococcales | Micrococcaceae | Arthrobacter | 0.00242 | 0.00016 |
| **587** | Micrococcales | Micrococcaceae | Unknown | 0.00126 | 0 |
| **609** | Rubrobacterales | Rubrobacteriaceae | Rubrobacter | 0.00015 | 0.00058 |
| **52408** | Rhizobiales | Rhizobiaceae | Rhizobium | 0.05153 | 0.05187 |
| **52411** | Rhizobiales | Rhizobiaceae | Rhizobium | 0.02335 | 0.02589 |

Figure S1


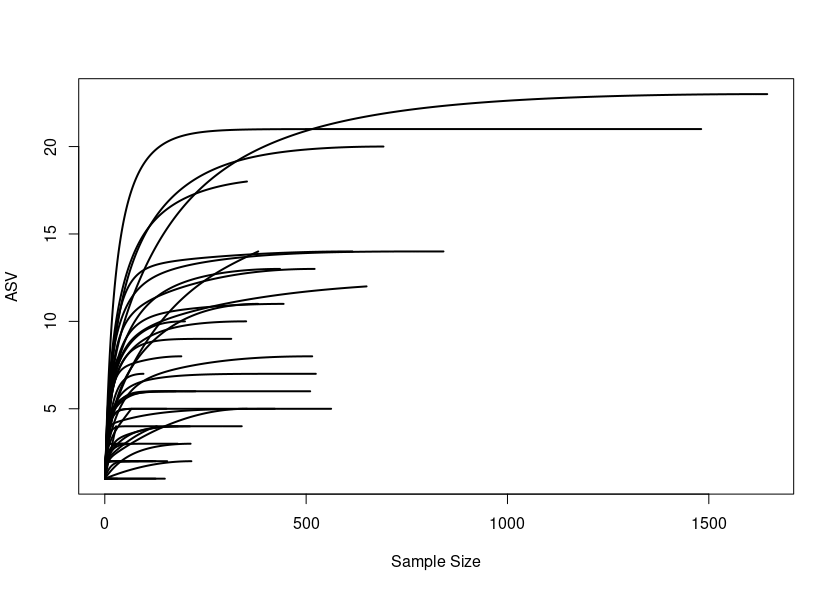


Root samples


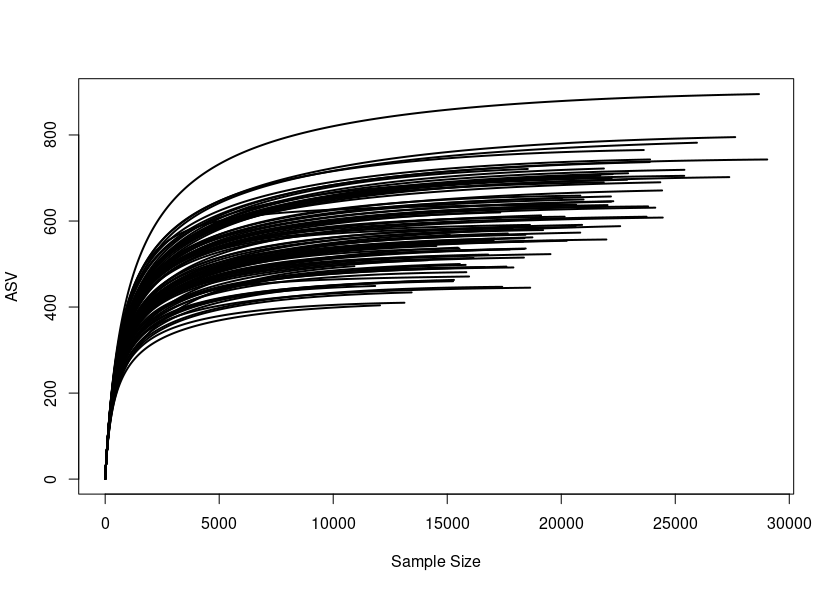


A

Soil samples

Figure S1. The Rarefaction curves for Roots and Soil samples consistently leveled off, suggesting that the sampling effort was sufficient.

Figure S2.


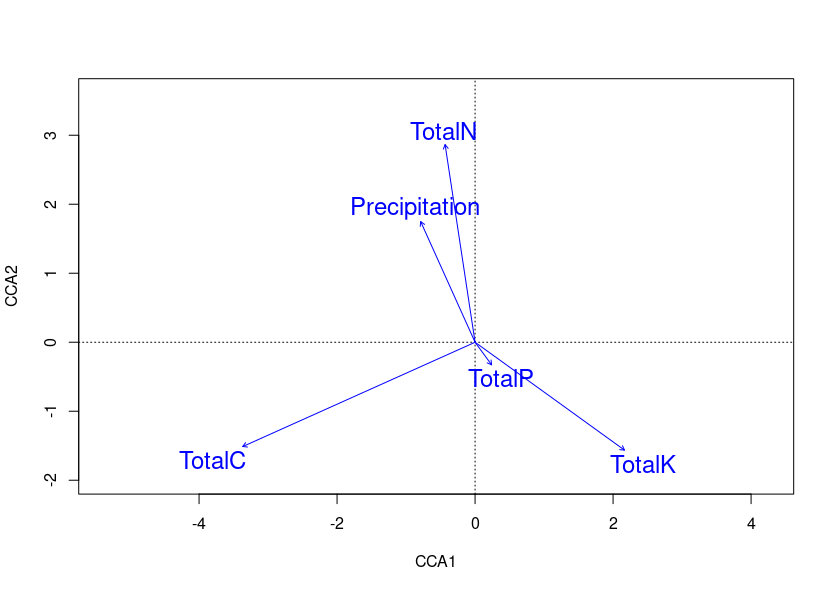


Figure S2. The CCA axis represents the ASV-level quantitative measurements for each soil sample.

Figure S3.


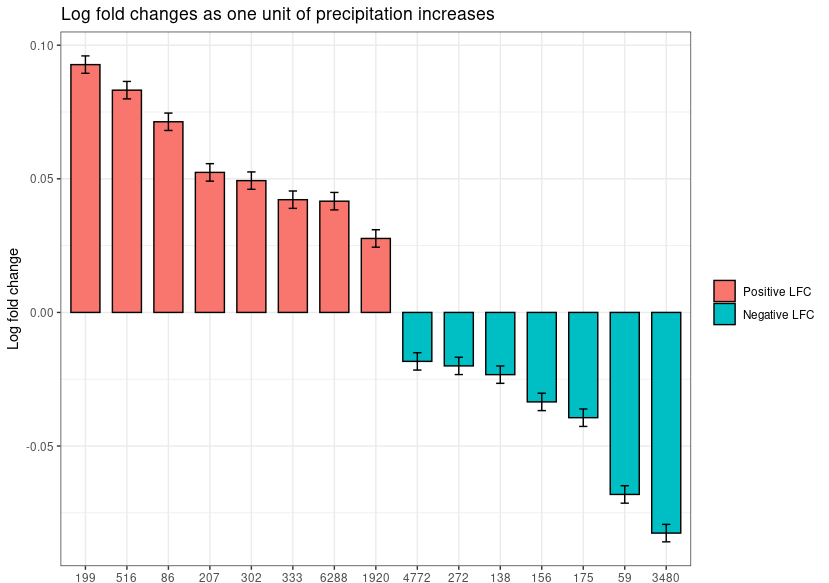


**Figure S3.** Taxa with significant log fold changes in their differential abundances when precipitation is increased by one unit, identified through the ANCOM method. It is noteworthy that none of these ASVs passed the sensitivity analysis test, suggesting a potential bias in the results due to the introduction of pseudo-counts.
